# COVID-19 isolation/quarantine rules in home care patients

**DOI:** 10.1017/S0950268822001844

**Published:** 2022-12-05

**Authors:** Beatrice Gasperini, Donatella Sarti, Tommaso Rondina, Margherita Moretti, Gilda Pelusi, Chiara Peconi, Emilia Prospero

**Affiliations:** 1 Department of Biomedical Sciences and Public Health, Section of Hygiene, Preventive Medicine, and Public Health, Università Politecnica delle Marche, Ancona, Italy; 2 Azienda Ospedaliera Ospedali Riuniti Marche Nord, Fano, PU, Italy; 3 School of Nursing Science, Università Politecnica delle Marche, Ancona, Italy

**Keywords:** Compliance rules, COVID-19, home isolation

## Abstract

The spread of Severe Acute Respiratory Syndrome Coronavirus 2 new variants increased the number of subjects in home isolation and quarantine. The aim of this study was to assess the compliance with coronavirus disease 2019 home isolation rules for 32 subjects in home care in Marche Region, Italy. The results showed that subjects in home isolation were better informed about isolation rules (*P* = 0.007) than those who were in quarantine. They had lower educational level (*P* < 0.001) and none/single income (*P* < 0.001) and higher rate of clinical manifestation. The education for a safe quarantine should be strengthened widely, especially among disadvantaged subjects.

## Introduction

Italy was the first European country to be severely affected by coronavirus disease 2019 (COVID-19) at pandemic onset. Lombardy was the Italian region with the highest number COVID-19 cases, followed by Emilia Romagna, Piedmont, Tuscany and Marche Region on 30 March 2020 [1, 2].

An effective COVID-19 transmission control depended on the monitoring and control of home isolation and quarantine through preventive and educational interventions [3, 4]. The fast evolution of COVID-19 pandemic required the activation of home isolation and quarantine measures for asymptomatic/mild symptomatic or exposed to COVID-19 subjects [5, 6]. The home isolation was indicated when subjects could be followed up and cared at home, respecting isolation rules. Quarantine was needed for subjects who were not infected, but had a close contact with a COVID-19 positive patient.

Isolating rules included staying in a separate bedroom, having access to food resources and consume them alone, washing hands frequently, maintaining social distancing and wearing a face mask when distancing was not possible and during care procedures. Previous studies reported a poor adherence to self-isolation rules and the need for better information about isolation/quarantine protocols from public health officials [7]. Home health care workers care for played an important role in supporting patients with confirmed and suspected COVID-19 who remain at home [8, 9].

Italian Nursing Home Service (NHS) provided a wide range of healthcare services at patient's home and assisted patients with COVID-19 during the pandemic. The aim of this study was to assess the compliance with the international isolation rules [1, 3, 4] for a proper home isolation and quarantine for COVID-19 of subjects in the home care of NHS.

## Method

This was a cross-sectional observational study conducted in Marche Region (Italy), among patients in home isolation or quarantine for COVID-19 and their relatives under the NHS in Ancona and Pesaro Urbino provinces, between 7th of May and 30th of June 2020. Some of those already received NHS visits before the COVID-19 spread, whereas others started to be assisted by NHS after COVID-19 diagnosis or exposure.

Nurses gave the adequate recommendation on isolation/quarantine to patient during their first home visit and personally verified the isolation/quarantine conditions, using a semi-structured questionnaire.

Instructions to clarify informed consent were provided to participants and all participants signed the informed consent. Responders were guaranteed anonymity. Inclusion criteria were being in home isolation or quarantine for COVID-19 between 7th of May and 30th of June 2020. No exclusion criteria were applied.

The questionnaire investigated variables that were in accordance with the isolation rules of Italian National Institute of Health recommendations [1]. The questionnaire assessed patient's and caregiver's (if any) age, gender and educational degree (< 9 years or ≥9); the number of family incomes (none/single, or multi-income per month), presence of households with increased risk of contagion (people older than 70 years and/or chronically ill).

Data collected included: the patient's clinical history, result of the Severe Acute Respiratory Syndrome Coronavirus 2 (SARS-CoV2) rhino-pharyngeal swab, presence of COVID-19 symptoms, presence of a caregiver (relative or paid assistant) providing care and ensuring proper isolation was explored, presence of personal protective equipment (PPE) and antiseptic and their correct use. Questionnaire items were reported in Appendix 1. The questions were made to subjects and their relatives. The general opinion of the home care nurses about the adherence to isolation/quarantine rules was recorded at the end of the questionnaire.

Mean and standard deviation (s.d.) for continuous variables and frequency and percentage for categorical variables were computed to describe the sample. Subjects were divided in home isolation and quarantine according to the result of the SARS-CoV2 rhino-pharyngeal swab (positive or negative/not performed). Data analysis was performed using SPSS^®^ version 25 (SPSS Inc., Chicago, IL, USA) for Windows^®^. The level of significance was set at 0.05.

## Results

A total of 33 subjects and their caregivers were invited and 32 of them accepted to participate to the study (acceptance rate 97%). The number of subjects in home isolation for a positive SARS-CoV2 rhino-pharyngeal swab was 13 (41%) and 19 (60%) subjects were in quarantine. Characteristics of the sample were reported in Table 1. Mean age was 71 ± 13.6 years (range 39–91), 46.9% were females and 75% were already under care of home care services before the COVID-19 pandemic; 8 (25%) subjects were discharged from hospital. Most of subjects had a caregiver (81.2%), and 28.1% lived with a family member at increased risk of infection. Subjects' educational level was lower for subjects in isolation than for those in quarantine (61.5% *vs* 26.3%, *P* < 0.001). Subjects in isolation had none/single income (86.4% *vs* 31.6%, *P* < 0.001). The main comorbidity was cardiovascular in both groups (46.2% and 52.6% respectively, *P* = 0.750).

**Table 1.** Socio-demographic characteristics of the sample

	Total *N* = 32	Home isolation *N* = 13	Quarantine *N* = 19	
	*n* (%)	*n* (%)	*n* (%)	*P*
Age (mean ± s.d.)	71.16 (13.6)	67.7 (15.9)	73.5 (9.8)	0.291
Females	15 (46.9)	6 (46.2)	9 (47.4)	0.946
Subjects already under the HCN	24 (75.0)	8 (61.5)	16 (84.2)	0.146
Subjects discharged from hospital	8 (25)	5 (38.5)	3 (15.6)	0.146
Caregiver	26 (81.2)	11 (84.6)	15 (78.9)	0.687
Education < 9 years				
Subject	13 (40.6)	8 (61.5)	5 (26.3)	**<0**.**001**
Caregiver	9 (28.1)	4 (30.8)	5 (26.3)	0.481
Income				
None/Single	17 (55.2)	11 (84.6)	6 (31.6)	**<0**.**001**
Multi-income	15 (46.9)	2 (15.4)	13 (68.4)	**<0**.**001**
Hospitalisation (last 3 months)^a^	9 (28.1)	5 (38.5)	4 (21.1)	0.282
Cardiovascular disease	16 (50)	6 (46.2)	10 (52.6)	0.480
Chronic renal failure	3 (9.4)	2 (15.4)	1 (5.3)	0.999
Cancer	6 (18.8)	2 (33.3)	4 (66.7)	0.654
Diabetes	2 (6.3)	1 (5.3)	1 (7.7)	0.999
Dementia	2 (6.3)	1 (5.3)	1 (7.7)	0.484
No chronic diseases	7 (21.9)	6 (46.2)	1 (5.3)	0.083

a Non COVID-19 related hospitalisation.

Comparison between subjects in home isolation or quarantine.

Subjects in isolation declared more often to have anosmia, ageusia, dyspnoea, pharyngodinia (69.4% *vs* 4%, *P* < 0.001), whereas subjects in quarantine had general malaise (16.1% *vs* 28%, *P* = 0.042). There were no differences as concerning fever, cough and diarrhoea between the two groups (Table 2).

**Table 2.** Clinical symptoms of COVID-19

	Total *N* = 32	Home isolation *N* = 13	Quarantine *N* = 19	
	*n* (%)	*n* (%)	*n* (%)	*P*
Fever	19 (34.5)	10 (32.3)	9 (36)	0.581
General malaise	12 (21.8)	5 (16.1)	7 (28)	**0**.**042**
Cough	9 (16.3)	4 (12.9)	5 (20)	0.176
Anosmia, Ageusia, Dyspnoea, pharyngodinia	10 (31.3)	9 (69.2)	1 (4)	**<0**.**001**
Diarrhoea	3 (5.5)	2 (6.4)	1 (4)	0.445

Comparison between subjects in home isolation or quarantine.

About 15% of subjects had a caregiver who was not a family member. Caregiver was able to understand the hygienic rules and the correct use of PPE in both isolation and quarantine subjects (*P* = 0.496). The majority (<80%) of subjects were able to have meals.

The 75% of the sample knew the isolation rules, with a significant difference between patients in isolation and quarantine (100% *vs* 57.9%, *P* = 0.007). Face mask (100% and 89.5%, *P* = 0.227) and antiseptics (84.6% and 73.7%, *P* = 0.222) were present in most houses. A greater number patients in quarantine than those in isolation had a house of their property (89.5% *vs* 61.5%, *P* = 0.05)

In the opinion of home nurses, an adequate home isolation was adopted in 12 subjects (92.3%) in home isolation and in 12 (63.2%) of subjects in quarantine (*P* = 0.061). (Table 3).

**Table 3.** Social support, house characteristics, presence of PPE and antiseptic, possibility of maintaining relationships using technological tools

	Total *N* = 32	Home isolation *N* = 13	Quarantine *N* = 19	
	*n* (%)	*n* (%)	*n* (%)	*P*
Non-family member caregiver	5 (15.6)	2 (15.4)	3 (15.8)	0.975
Ability to provide meals	28 (87.5)	11 (84.6)	17 (89.5)	0.683
Caregiver able to understand hygiene rules	28 (87.5)	12 (92.3)	16 (84.2)	0.496
Knowledge of isolation rules	24 (75)	13 (100)	11 (57.9)	**0**.**007**
PPE correct use	30 (93.8)	13 (100)	17 (89.5)	0.482
Smartphone	15 (46.9)	6 (46.2)	9 (47.4)	0.946
Video call	13 (40.6)	6 (46.2)	7 (36.8)	0.598
PC	8 (25)	5 (38.5)	3 (15.8)	0.148
Own home	25 (78.1)	8 (61.5)	17 (89.5)	**0**.**05**
Face masks	30 (93.8)	13 (100)	17 (89.5)	0.227
Antiseptics	25 (78.1)	11 (84.6)	14 (73.7)	0.222
Adequate home isolation	24 (75)	12 (92.3)	12 (63.2)	0.061

PPE, personal protective equipment.

Comparison between subjects in home isolation or quarantine.

## Discussion

This study aimed to verify the compliance with the rules for a proper home isolation and quarantine for COVID-19 in subjects under nurses' home care.

Subjects in home isolation were better informed about isolation rules (*P* = 0.007) than who were in quarantine. They had lower educational level (*P* < 0.001) and none/single income (*P* < 0.001). Home care nurses considered not adequate the isolation condition in 25% of the whole sample and in almost 40% of patients in quarantine.

The evaluation of patient's home setting according to infection prevention and control criteria was conditional to the possibility to maintain at home subjects in isolation or quarantine. When these rules of home isolation could not be respected, the subjects should be isolated in non-traditional facilities, such as dedicated hotels, where they could remain until symptoms resolution and the results of laboratory tests for SARS-CoV2 become negative [10].

A systematic review reported that there is mixed evidence on the correlation between demographic and employment characteristics of subjects in home isolation and their adherence to home isolation protocol [11]. This suggests that an educational programme could be effective in general population to improve adherence to isolation and quarantine regulations.

Subjects in isolation had a better knowledge of the isolating rules. This may be due to the general practitioner or health care providers had spent more time to explain them the rules for a proper isolation. Another explanation was related with an increased awareness of infected subjects about a correct isolation and knowledge of the importance to respect isolation rules to prevent the infection of their relatives. Subjects in quarantine had a lower perception of the risk for a possible familiar contagion than those in isolation after a diagnosis of SARS-CoV2 infection.

Home care nurses considered not adequate the isolation condition in 25% of the whole sample and in almost 40% of patients in quarantine. This was in accordance with the current evidence suggesting that the adherence to self-isolation is generally low. Sehgal and colleagues in their study reported a similar result on the feasibility of separate rooms for isolation and quarantine for housing units in the United States. They showed that solation or quarantine was impossible in 20.8% of all U.S. residential units, because they lacked sufficient bedrooms, bathrooms or both [12]. The adherence to full self-isolation was 42.5% in UK and only 11% of close contacts were quarantined [13].

Our findings suggest the need for higher knowledge on isolation rules for subjects in quarantine. For this reason, it is important that COVID-19 positive subjects adopt isolation rules and that subjects in quarantine respect these rules not to be infected. Precautionary measures were often taken when it was too late.

Several studies show home isolation efficacy in reducing COVID-19 incidence and mortality [14–16]. In our study, it emerges that the level of knowledge on prevention measures effective to reduce SARS-CoV2 transmission are reached when it is too late: patients in isolation reach the highest level of knowledge compared to those in quarantine and the difference between the two groups is statistically significant.

Even if the clinical presentation of COVID-19 has changed since the beginning of the pandemic, there are some population groups with an increased risk of unfavourable outcomes. Health disadvantage due to weaker socio-economic status increases this risk further. For this reason, the identification of these frail subjects should be improved to adopt specific interventions. For future pandemics requiring isolation and quarantine, it is mandatory for health professionals to focus on educational programmes targeting people with a low socio-demographic level, who are at increased risk to get infected by family members. The main limitations of our study are the small number of subject enroled and the context where it was carried out, indeed, NHS could be quite different among countries.

NHS is a care formula dedicated to the elderly and all people who are not self-sufficient and is regulated by the National Health Service. This service is carried out at patient's home, for this reason it is useful for monitoring isolation/quarantine adherence. This is consistent with surveillance strategies adopted by other countries, supporting the generalisability of our findings. Broadly, local public health authorities or private sector staff coordinate isolation/quarantine checks with regular or random checks conducted in person or by telephone, and digital surveillance technologies [17].

## Conclusions

Subjects in home isolation were more informed than who was in quarantine about the isolation rules. The education for a safe quarantine should be widely strengthened. Home care nurses may be the most indicated health care providers to assess the compliance with a safe quarantine or home isolation for COVID-19 and promote a proper home isolation or quarantine, having the trust of patients and a significant role in home care.

## Data Availability

The datasets generated during and/or analysed during the current study are available from the corresponding author on reasonable request.

## Supplementary material

For supplementary material accompanying this paper visit https://doi.org/10.1017/S0950268822001844. click here to view supplementary material
