# Supplementary material for: COVID-19 isolation/quarantine rules in home care patients
Source: Epidemiol Infect. 2022 Dec 5;150:e206. doi: 10.1017/S0950268822001844 (PMC9767896; doi:10.1017/S0950268822001844)
Supplement: Supplementary file 1 [file S0950268822001844sup001.docx]

**Appendix 1) Questionnaire on home isolation/quarantine conditions for COVID-19**

**Age ____ Gender**___M □ F □

**The Patient Lives Alone** YES □ NO □

**Subjects already under the HCN** YES □ NO □

**Patient’s Educational Level:**

□ Elementary School □ Middle School □ High School

**Caregiver’s Educational Level:**

□ Elementary School □ Middle School □ High School

**Income:** Single□ Mutiple □ None **□**

**Hospitalization from 31 January 2020** YES □ NO □

**COVID-19 swab result:** □ Positive (Date ______) □ Negative □ Not Made

**Patient Symptomatic for COVID 19** (even if swab negative or not carried out) **Symptoms:**

Fever □ Dyspnea □ General Disease □ Cough □ Expectorate □ Cold □

Confusional State □ Sore Throat □ Other________________________

**Chronic diseases**

Cardiovascular Yes □ No □ Chronic obstructive pulmonary disease Yes □ No □

Kidney chronic disease Yes □ No □ Diabetes Yes □ No □

Cancer Yes □ No □

**Other Chronic Diseases ____________________________________________________________**

**Number Of Families (Cohabitants) _____**

**Number Of Rooms______**

**Owned House** Yes □ No □

**House For Rent** Yes □ No □

**Size Of The House ( Approximately Square Meters ) _______**

**House Developed On Multiple Floors** Yes □ No □

**Adequate General Hygienic Conditions Of The House**

(cleaning, ventilation, brightness) Yes □ No □

**Presence And Use Of Antiseptics** Yes □ No □

If Yes Presence Of Masks Yes □ No □

**Knowledge Of Insulation Instructions:** Yes □ No □

(disposal of unsorted waste, use of disinfectants for washing the patient's clothes, etc.)

**Possibility Of Keeping The Patients In Isolation** Yes □ No □

(single room, bathroom and separate toilet)

**Non-Family Caregiver Present** Yes □ No □

**Caregiver able Apply hygenic standards**

(family member or not) Yes □ No □

**Possibility of Providing Food and Resources of First Necessity**

(including material for suitable disinfection) Yes □ No □
